# A Printed Xi-Shaped Left-Handed Metamaterial on Low-Cost Flexible Photo Paper

**DOI:** 10.3390/ma10070752

**Published:** 2017-07-05

**Authors:** Farhad Bin Ashraf, Touhidul Alam, Mohammad Tariqul Islam

**Affiliations:** Department of Electrical, Electronic and Systems Engineering, Universiti Kebangsaan Malaysia, Bangi, Selangor D.E. 43600, Malaysia; farhadbinashraf@siswa.ukm.edu.my (F.B.A); touhid13@siswa.ukm.edu.my (T.A.)

**Keywords:** double negative, flexibility, inkjet printing, metamaterial, nanoparticle ink technology, paper substrate

## Abstract

A Xi-shaped meta structure, has been introduced in this paper. A modified split-ring resonator (MSRR) and a capacitive loaded strip (CLS) were used to achieve the left-handed property of the metamaterial. The structure was printed using silver metallic nanoparticle ink, using a very low-cost photo paper as a substrate material. Resonators were inkjet-printed using silver nanoparticle metallic ink on paper to make this metamaterial flexible. It is also free from any kind of chemical waste, which makes it eco-friendly. A double negative region from 8.72 GHz to 10.91 GHz (bandwidth of 2.19 GHz) in the X-band microwave spectra was been found. Figure of merit was also obtained to measure any loss in the double negative region. The simulated result was verified by the performance of the fabricated prototype. The total dimensions of the proposed structure were 0.29 *λ* × 0.29 *λ* × 0.007 *λ*. It is a promising unit cell because of its simplicity, cost-effectiveness, and easy fabrication process.

## 1. Introduction

Metamaterials are artificial media with properties that are not readily available in nature. They are electromagnetic periodic structures in which periodicity is smaller than the wavelength of the material. Researchers are interested in this field because of its extraordinary electromagnetic properties. Metamaterials can simultaneously show negative permittivity and permeability, which make them different from other materials. The electromagnetic constitutive parameters lie in their construction, and not in the composite of the materials. Even so, some materials show negative permittivity like gold, silver, etc., but the permeability is very difficult to determine in general. The Russian physicist Victor Veselago is the one who first gave the theoretical explanation of materials as having left-handed characteristics in 1968 [1]. In 2001, Shelby et al. showed the negative index of refraction using a two dimensional array of repeated unit cells that combined with wire strips and split-ring resonators [2]. Metamaterials are mostly designed on a Flame Retardant-4 (FR-4) substrate. Advanced technologies of the 21st century all aim at compact, lightweight, and cost-effective properties. These circumstances encourage researchers to go for low cost and easily fabricated substrates, such as paper. Another advantage of using paper is its flexibility. This fabrication technology can provide researchers with a new field of investigation. With its unusual properties, paper can be applied in a flexible ultra-wideband antenna [3], flexible frequency selective [4], beam-tilting [5], filter design [6], electromagnetic cloaking [7], sensors [8], etc. Liu et al. proposed a modified circular electric resonator [9], where it can only achieve a 1-GHz bandwidth of the negative refractive index. A non-planar metamaterial that has double negative characteristics, from 9.2 GHz to 10.1 GHz, with 900 MHz of double negative (DNG) bandwidth [10], is less than half of the proposed structure (bandwidth of 2.19 GHz) for X-band applications. Islam et al. [11] proposed a metamaterial with 0.7 GHz bandwidth of negative refractive index in the X-band. The dimensions of the metamaterial unit cell were 30 × 30 × 1.6 mm^3^. In reference [12], a “Z-shaped” resonator having a 2.17 GHz double negative region using the FR-4 substrate was presented, which was a good achievement, but the substrate flexibility and fabrication hazards were not within the confines of the experiment. Most of the metamaterial designed were considered on hard substrates, such as FR-4 materials [13] and silicon [14]. As these materials are inflexible, metamaterials are bound to planar surfaces. Joshi et al. [15] proposed a metamaterial embedded wearable rectangular antenna, and were able to get negative permeability only in the frequency range of 8.35 GHz to 8.7 GHz. Tenggara et al. [16] also proposed a metamaterial in the THz range, and they used electro hydrodynamic jet printing technology. A broadband flexible metamaterial absorber was proposed in Reference [9] using a flexible polyethylene terephthalate (PET) substrate where the authors found a wide absorptivity from 7.9 to 18.74 GHz. In Reference [17], Kim et al. presented an inkjet-printed Electromagnetic Bandgap (EBG) array on a paper substrate for wearable applications.

This study presents an electrically-small metamaterial unit cell, based on modified SRR and CLS, using inkjet printing technology. Photo paper was used as a substrate and a metallic nanoparticle ink was used as a conductive radiating element. Some printed radio frequency identification (RFID) antennas also used paper as a substrate [18]. Metamaterials should be bendable to nonplanar or curved surfaces for attachment in wearable wireless communication. With applications that use appropriate coatings, paper can easily obtain a low surface profile, which can allow the utilization of direct writing methods like inkjet printing, as a replacement for comparatively-expensive wet etching practices, and also avoid the typical soldering of lumped resistors. The dielectric constant of paper is like the rigid substrate used in literature. It gives wide negative refractive index (NRI), even with a lower thickness. Miniaturization of the unit cell was achieved by increasing the capacitance and inductance. The electric and magnetic response can also be tuned by varying the length of the CLS. The structure has simultaneous negative permittivity and permeability, which produce a wide double negative band to any polarized incident wave. The simulated S-parameters are obtained by using Computer Simulation Technology (CST) Microwave Studio. A prototype was printed and measured with a waveguide measurement setup. 

## 2. Design of the Printed Unit Cell

**Figure 1:**
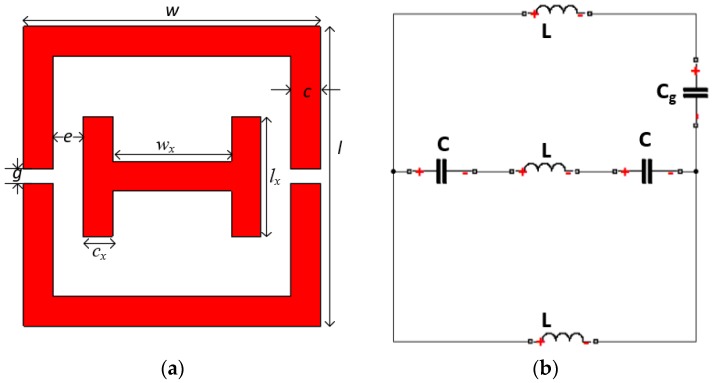
Layout of the proposed unit cell: (**a**) schematic view; (**b**) the equivalent circuit model.

A 12 × 12 mm^2^ unit cell of combined SRR and CLS is shown in Figure 1a. The paper substrate, where the printed structure has a relative permittivity of *ε* = 3.2, and is 0.27 mm in thickness [19]. The metallic ink is 0.0175 mm thick, which is used as a conductor. The unit cell configurations are shown in in Table 1. The losses are the restricting factors in practical applications.

The modified SRR acts as an artificial magnetic dipole. This magnetic dipole moment produces a frequency in the form of [20]:

(1) $$\mu (\omega )=\mu_{0}(1-\frac{\omega^{2}{}_{pm}}{\omega (\omega -j\Gamma_{m})})$$

and the CLS produces a dielectric response. This response creates an electric dipole moment, which produces a plasmonic-type of permittivity frequency in a function of:

(2) $$\varepsilon (\omega )=\varepsilon_{0}(1-\frac{\omega^{2}{}_{pe}}{\omega (\omega -j\Gamma_{e})})$$

where, Γ*_m_* and Γ*_e_* are damping coefficients and *µ*_0_ and *ɛ*_0_ are permeability and permittivity of free-space, respectively. The proposed structure shows the DNG band when the magnetic plasma frequency (*ω_pm_*) and the electric plasma frequency (*ω_pe_*) are greater than *ω*.

## 3. Methodology

The proposed structure has inductance L and capacitance C components, as shown in Figure 1b. The equivalent circuit model is the simplest LC resonator with a resonant frequency of:

(3) $$f=\frac{1}{2\pi \sqrt{LC}}$$

The resonance frequency depends on *L* and *C* of the resonator. By changing the geometry of the structure, they can be shifted. The inductance *L* are formed by the metal loop, and the capacitance *C* are formed by the gap between them. The couplings between electric fields and gaps are responsible for electric resonances and the coupling between magnetic fields and loops are responsible for magnetic resonance.

**Figure 2:**
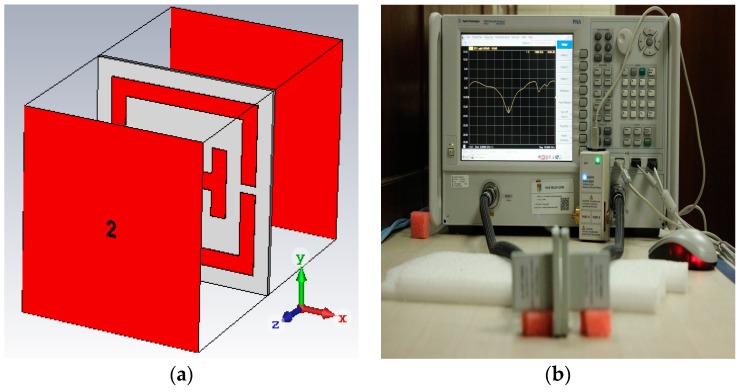
(**a**) Floquet port setup; (**b**) measurement setup.

The finite element method (FEM) that is based on the CST Microwave Studio was used to design and analyze the scattering parameters of the given structure. As shown in Figure 2a, the perfect electric conductor (PEC) and the perfect magnetic conductor (PMC) were applied in the *x*- and *y*-axis, respectively. The incident wave propagates in the direction of the *z*-axis by two waveguide ports. The tetrahedral mesh was used in the simulation. For the measurement process, an Agilent N5227A performance network analyzer (PNA) (CA, USA) has been used. An Agilent N4694-60001 electronic calibration module is also used to calibrate the PNA. A set of WR112 waveguide to coaxial adapter is connected to the PNA, having a dimension of 48 × 48 × 41.4 mm^3^. The prototype was placed between the two microwave waveguide ports, as shown in Figure 2b. 

According to [21,22,23], effective parameters have been extracted by using the complex S_11_ (reflection coefficient) and S_21_ (transmission coefficient) data.

(4) $$S_{11}=\frac{R_{01}(1-e^{i2nk_{0}d})}{1-R^{2}{}_{01}e^{i2nk_{0}d}}$$

(5) $$S_{21}=\frac{(1-R^{2}{}_{01})e^{ink_{0}d}}{1-R^{2}{}_{01}e^{i2nk_{0}d}}$$

where, $R_{01}=z-1/z+1$.

For a plane incident wave, the relative impedance *z* and effective refractive index *η* can be obtained by solving Equations (4) and (5):

(6) $$z=\pm \sqrt{\frac{{\left(1+S_{11}\right)}^{2}+S^{2}{}_{21}}{{\left(1-S_{11}\right)}^{2}+S^{2}{}_{21}}}$$

(7) $$e^{ink_{0}d}=X\pm i\sqrt{1-X^{2}}$$

where, $X=1/2S_{21}(1-S^{2}{}_{11}+S^{2}{}_{21})$.

(8) $$\eta =\frac{1}{k_{0}d}[\{[\ln(e^{ink_{0}d})]+2m\pi \}-i[\ln(e^{ink_{0}d})]]$$

where *m* is an integer that defines the branch index of η. The effective permittivity *ε* and permeability *μ* are then obtained, using:

(9) $$\varepsilon =\frac{\eta }{z}$$

(10) μ=ηz

## 4. Inkjet Printing Process

**Figure 3:**
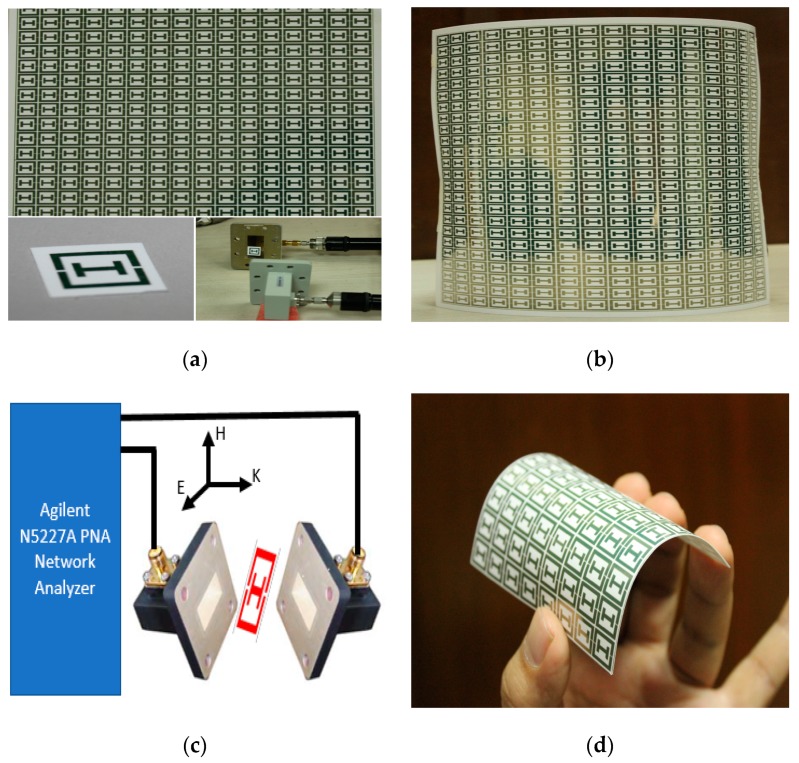
(**a**) Inkjet-printed metamaterial with waveguide port; (**b**) array of the prototype; (**c**) measurement setup; (**d**) flexible metamaterial prototype.

The photo papers from Epson are being used in many articles for inkjet printing [19]. A Brother MFC-J430W printer (Brother, Aichi Prefecture, Japan) was used for printing the structure, as shown in Figure 3a,b. A silver metallic ink (LC12M) from AgIC (Tokyo, Japan) was used. The main feature of this ink is that it dries in a few seconds. It has a working temperature from −20 to 70 °C. The shelf life of the ink is about one year. As the structure is single sided, it is simple and easy to print. There are no complications regarding the ground plane, like in other metamaterials.

To verify the performance of the proposed structure, shown in Figure 3b, a free space measurement setup has been adopted. The measurement is conducted in an anechoic chamber with two horn antennas (1 to 18 GHz) placed 1 m apart to satisfy the far-field condition; the array prototype structure is positioned between them by a sample holder. The chamber is surrounded by a wedge tapered absorber and the horn antennas are in the same plane according to the simulation geometry, to allow the wave to propagate over the prototype. The horn antennas are connected by the Agilent N5227A microwave network analyzer to measure the transmission coefficient.

## 5. Results and Discussion

**Figure 4:**
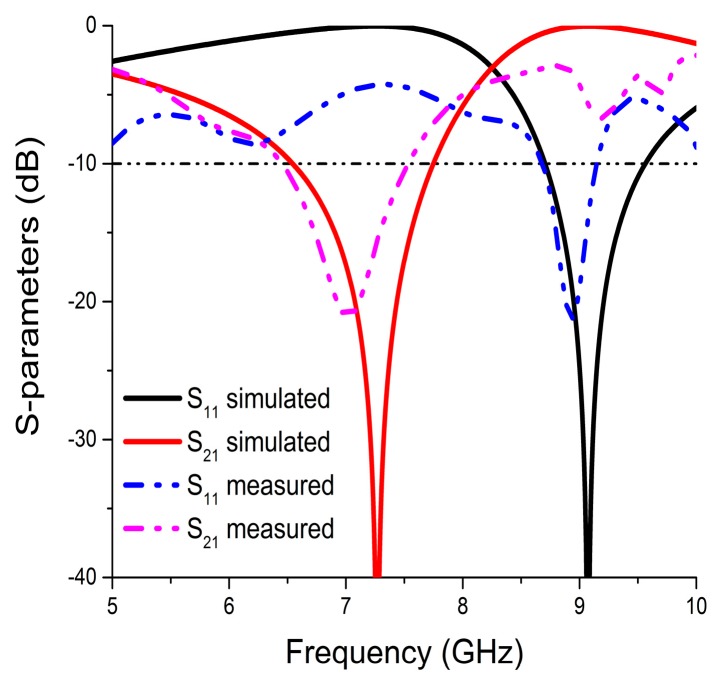
Simulated and measured S-parameters of the printed resonator.

**Figure 5:**
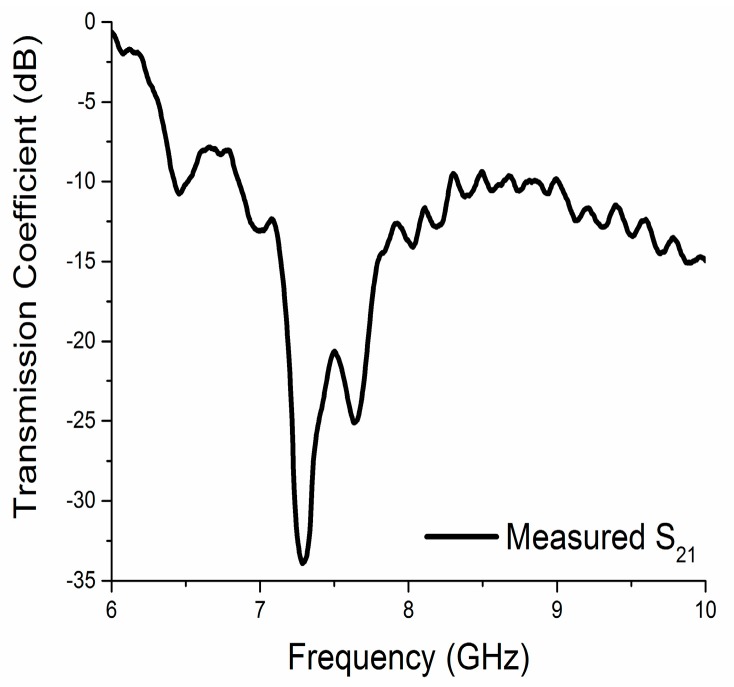
The measured transmission coefficient of the array prototype.

The simulation and measured results of the reflection coefficient (S_11_) and the transmission coefficient (S_21_) are shown in Figure 4. The simulated unit cell exhibits bandwidths (S_21_ < −10 dB) of 1.25 GHz (6.51–7.76 GHz) and (S_11_ < −10 dB) 0.88 GHz (8.69–9.57 GHz) that are centered at 7.26 GHz and 9.1 GHz, respectively. Finally, the fabricated prototype of the unit cell exhibits transmission coefficient bands (S_21_ < −10 dB) at 6.41 to 7.54 GHz and a reflection coefficient (S_11_ < −10 dB) of 8.69 to 9.15 GHz that are centered at 7.26 GHz and 9.1 GHz, respectively. The measured transmission coefficient of the array prototype is depicted in Figure 5. It showed a bandwidth of 1.42 GHz (6.85–8.27 GHz), which was below −10 dB. The printed prototype illustrated a slight disagreement between the measured and the simulated results. The reasons behind this dissimilarity are fabrication tolerance and measurement tolerance.

**Figure 6:**
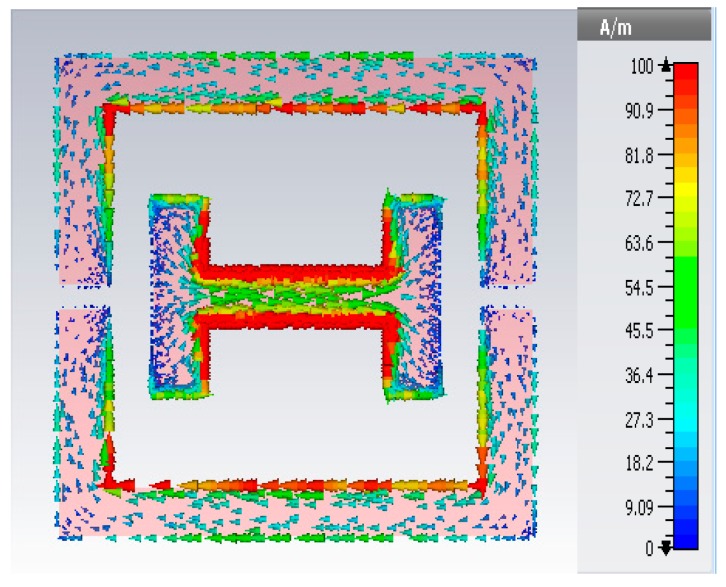
Surface current distribution at 8.8 GHz.

The surface current at 8.8 GHz were shown to explain how the magnetic response and electric response were exhibited, as shown in Figure 6. According to Faraday’s law, a time changing magnetic field that is polarized perpendicularly to the plane of the MSRR induced a circulating current. Next, the splits of the MSRR acts as a capacitor by storing energy across the gap because of the circulating current. Two parallel current paths in the outer side of the CLS provide a wide electric plasma frequency. The plasma frequency exhibits negative permittivity because of free electrons in the arm that screen external electromagnetic radiation. The current loop in the two arms of the modified split ring resonator form an LC resonant circuit, which results in an out of phase or exhibits a negative response when the circulating current lags the external magnetic field. 

**Figure 7:**
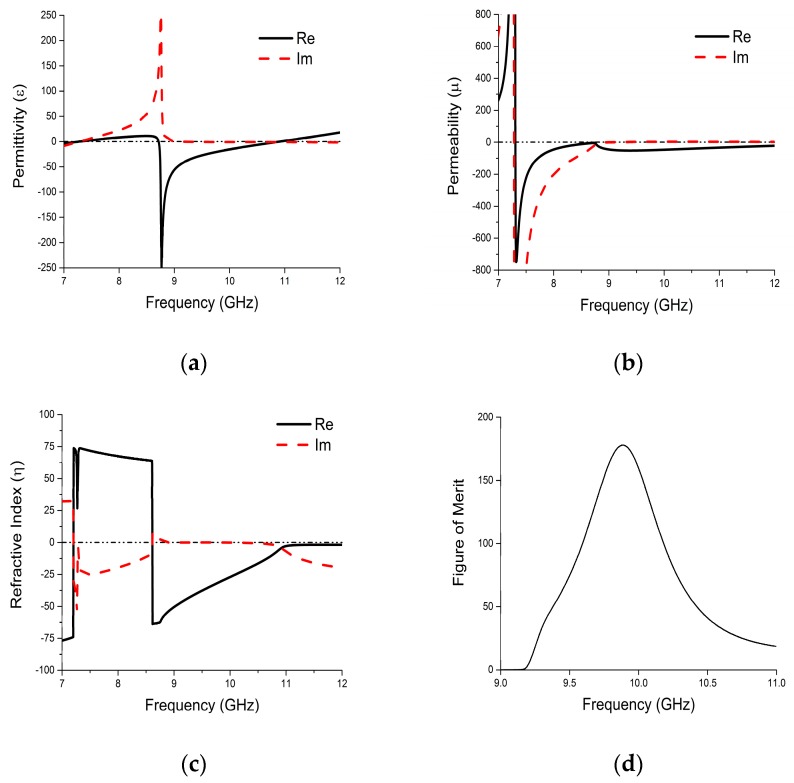
Extracted effective parameters: (**a**) effective permittivity; (**b**) effective permeability; (**c**) index of refraction; (**d**) figure of merit.

The effective relative parameters of the proposed metamaterial are depicted in Figure 7. There is a wide electric response for the CLS in Figure 7a, and a very wide magnetic response for the outer split-ring, as shown in Figure 7b. One of the electric responses is from 8.72 GHz to 10.9 GHz. The magnetic response has a bandwidth of 4.69 GHz. An ultra-wide double negative pass-band is formed when these two overlap. From Figure 7c, it can be seen that a 21% wide left-handed region has been achieved from 8.72 GHz to 10.9 GHz. The loss near resonance point is higher, as expected. The figure of merit (FOM) in Figure 7d demonstrates loss of the structure in the DNG band using an absolute value of real (*η*)/imaginary (*η*) [24].

**Figure 8:**
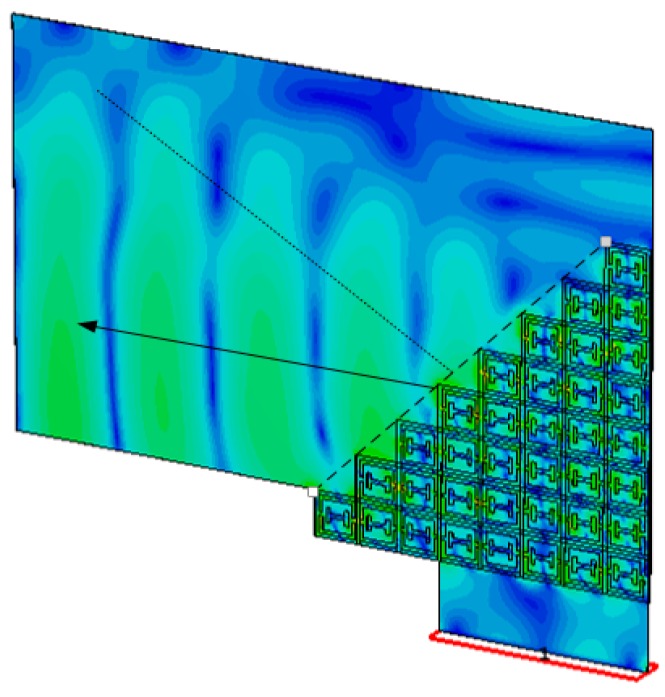
Magnitude of the e-field at 8.8 GHz.

To validate the presence of the refractive index, a wedge-shaped metamaterial was designed. It is a widely used method—used to verify the left-handed behavior on Snell’s law [25]. For the wedge-shaped metamaterial, as shown in Figure 8, eight unit cells in the *x*-axis and eight unit cells in the *y*-axis were placed. It has a staircase pattern in both the *x* and *y* directions, which sets the wedge angle to 45°. The DNG band of the proposed unit cell is between 8.72 and 10.9 GHz. The magnitude of e-field is illustrated in Figure 8, which exhibits the index of refraction at 8.8 GHz. The straight line is the normal surface, and the arrow line shows the refracted waves.

From Table 2, a comparison of the proposed unit cell with an existing unit cell is shown to analyze the performance of the proposed metamaterial.

## 6. Conclusions

A Xi-shaped left-handed metamaterial composed with SRR and CLS has been presented. The use of the photo paper substrate material makes the structure easier to fabricate and also cost effective. Moreover, the flexibility of this material makes it more effective in the field of wearable technology. The symmetric structure exhibits about 21% negative refractive index bandwidth, which is considered as a wide left-handed material. The electric and magnetic response of the structure has been studied. According to simulated and measured results, the proposed structure could be a potential candidate for X-band applications like military radio communication, weather observation, and terrestrial communications, and the list goes on and on. It can also be used for breast tumor detection using a flexible wearable microwave imaging system.

## Figures and Tables

**Table 1:** Design parameters of the structure.

Parameters	Values mm	Parameters	Values mm
*w*	10	*c*	1
*l*	10	*w_x_*	4
*g*	0.5	*l_x_*	4
*e*	1	*c_x_*	1

**Table 2:** Comparison of the proposed unit cell with other unit-cells.

Ref. No.	Substrate	Negative Refractive Index Bandwidth	Metamaterial Type
[10]	FR-4	900 MHz	DNG
[11]	FR-4	700 MHz	DNG
[12]	FR-4	2.17 GHz	DNG
[15]	Polyester	350 MHz	SNG
Proposed unit-cell	Photo paper	2.19 GHz	DNG
